# Postnatal Identification of Trisomy 21: An Overview of 7,133 Postnatal Trisomy 21 Cases Identified in a Diagnostic Reference Laboratory in China

**DOI:** 10.1371/journal.pone.0133151

**Published:** 2015-07-15

**Authors:** Weiwei Zhao, Fan Chen, Menghua Wu, Shuai Jiang, Binbin Wu, Huali Luo, Jingyi Wen, Chaohui Hu, Shihui Yu

**Affiliations:** 1 KingMed Genome Diagnostic Laboratory, Guangzhou, China; 2 Department of Laboratory Medicine, University of Washington School of Medicine and Seattle Children’s Hospital, Seattle, WA, United States of America; Università degli Studi di Milano, School of Medicine., ITALY

## Abstract

This study describes the cytogenetic characteristics of 7,133 trisomy 21 (Tri21) identified from 247,818 consecutive postnatal cases karyotyped in a single reference laboratory in China for a period of 4 years. The average detection rate of Tri21 is 2.88% ranging from 3.39% in 2011 to 2.52% in 2014. The decreased detection rates over the years might reflect a possible impact of noninvasive prenatal testing applied rapidly in China and elective termination of affected pregnancies. 95.32% of the Tri21 karyotypes are standard Tri21, 4.53% are Robertsonian Tri21, and less than 1% are other Tri21 forms. There are more mosaic Tri21 in older children and adults, consistent with previous observations that clinical features in individuals with mosaic Tri21 are generally milder and easily missed during perinatal period. The male/female (M/F ratio) for the total 7,133 Tri21 cases and for the 6,671 cases with non-mosaic standard Tri21 are 1.50 and 1.53 respectively, significantly higher than the 0.93 for all the 247,818 cases we karyotyped, the 1.30 for the Down syndrome (DS) identified during perinatal period in China, and the 1.20 for the livebirth in Chinese population. In contrast, the mosaic standard Tri21 case has a significantly lower proportion of males when compared with the non-mosaic standard Tri21, indicating different underlying mechanisms leading to their formations. Opposite M/F ratios in different subtypes of ROB Tri21 were observed. A long list of rare or private karyotypes where Tri21 are concurrently present was identified. The large collection of Tri21 cases with a diversity of clinical findings and a wide age range allowed us to determine the frequency of the different karyotypes of Down syndrome in China, given the fact that this kind of national epidemiological data is lacking currently.

## Introduction

Trisomy 21 (Tri21), the cause of Down syndrome (DS), is the most common chromosome abnormality and the most common single known cause of intellectual disability in humans although approximately 80% of Tri21 pregnancies end in a miscarriage or intrauterine fetal demise, and only 20% may progress to term delivery [1,2]. The reasons causing Tri21 formation are not fully understood and maternal age is the most consistent biological factor associated with increased Tri21/DS prevalence and the major known reason leading to variable Tri21/DS prevalence ranging from 1/700-1/2,000 in different racial/ethnic populations investigated [3–16]. Standard Tri21 (47,XX,+21 or 47,XY,+21) have been consistently reported to accounts for 94–96% of all Tri21/DS cases in different racial/ethnic populations. However, in addition to the variable prevalence, proportions of rare Tri21 karyotypes derived from isochromosome 21, unbalanced Robertsonian translocations (ROB), and other chromosomal abnormalities involved in the formation of Tri21 vary widely. Higher proportions of males with Tri21/DS were observed in all populations, but wide variations of the male/female ratios (M/F ratio) exist in the literature [8,17–19]. Data related to the M/F ratios in some subtypes of Tri21, such as in mosaic Tri21 and different types of ROB Tri21, were available from few large scale investigations and were limited to Caucasian populations [8,17]. Postnatal Tri21/DS survivors share a long list of clinical findings such as intellectual disabilities, craniofacial features, congenital heart defects, leukemia, and others while the penetrance and expressivity for some of the clinical findings are highly variable in different racial/ethnic populations [1,2,20]. For example, compared with whites, atrioventricular septal defects (AVSD) in children with DS showed significant sexual and racial/ethnic differences with twice as many affected females and, twice as many blacks and half as many Hispanics. Biologically, besides maternal age, it was proposed that there are other biological factors, such as genetic variations, might have contributed to these differences although a long list of non-biological factors exists [4,20].

According to the “Report on birth defects in China, 2012” published by the Ministry of Health, China (http://www.gov.cn/gzdt/att/att/site1/20120912/1c6f6506c7f811bacf9301.pdf), (1) there are about 16 million newborns in 2011 in China, (2) of the 16 million newborns, approximately 900,000 of them (5.6%) were estimated to be affected by 23 types of monitored birth defects, (3) approximately 22.7% of the pregnancies nationwide were prenatally screened for DS, and (4) the prevalence of DS from 28^th^ gestation weeks to 7 days after birth is about 1/680. However, a recent publication by the National Center for Birth Defects reported a very low perinatal prevalence of DS in China, 1/5,025, including all DS of live birth, stillbirth, and terminated fetuses equal to or greater than 28 gestation weeks [11]. Chromosomal abnormalities including Tri21 could be only presumed to be one of the most important causes of the birth defects for the 900,000 affected newborns due to limited data availability. Even in the Unites States of America, in spite of widespread application of prenatal screening and diagnosis for DS, more than 85% of mothers who have children with DS first received the diagnosis postnatally [21]. Although the majority of postnatal DS cases can be identified based on clinical features, available data showed that 29–36% of clinically diagnosed or suspected DS were subsequently confirmed to be false positive [22,23]. Thus, cytogenetic characterization is essential not only to confirm the diagnosis but also to differentiate the subtypes of Tri21 for genetic counseling purpose since different subtypes of Tri21 are connected to different risks of recurrence as well as to reveal underlying genetic variations which might have contributed to the variations of Tri21/DS in different racial/ethnic populations. In this report, we shared our cytogenetic findings of 7,113 Tri21 cases identified in a single accredited laboratory in China.

## Materials and Methods

### Cases information

247,818 postnatal karyotypes were successfully performed from Jan., 2011 to Dec. 2014 in KingMed Genome Diagnostic Laboratory, a CAP and ISO 15189 accredited laboratory in Guangzhou, Guangdong province, China. Although more than 350 out of the 247,818 cases had ambiguous or unknown gender at the time of requisition for cytogenetic analysis, genotypic genders in the majority of them could be determined subsequently based on their karyotypes (genders were determined based on their karyotypes, not phenotypes), which left only 20 cases without assigned genders. Referral reasons for testing varied greatly. The majority of children referred for testing involved one or more of the following clinical findings: developmental delay, autism, dysmorphic features, seizures, or multiple congenital anomalies while the majority of adults referred for testing involved infertility, miscarriage, or offspring(s) with known chromosomal abnormalities. All adult participants in this study have provided their written consent by the participants or their caretakers. We obtained written consent for all minors/children enrolled in this study from their parents or guardians. This study was approved by the institutional review board of KingMed Genome Diagnostic Laboratory.

### Karyotyping method

Standard chromosomal analyses with trypsin-Giemsa banding were performed on routinely cultured peripheral blood lymphocytes. Twenty metaphases were analyzed for non-mosaic cells and sixty metaphases were analyzed for mosaic cells or suspected mosaic cases. Nomenclatures were assigned for each karyotype according to the international system for human cytogenetic nomenclature (ISCN 2013). Based on the presumed differences of the etiologies causing the formation of Tri21, a classification method of Tri21 karyotypes was proposed by Mutton et al. and this method was adopted in the current study with minor modifications [24].

### Statistical analysis

Chi square tests were used to do statistical analyses in this study. The M/F ratios for the total Tri21 as well as different subtypes of Tri21 were mainly compared with the M/F ratio for the total 247,818 postnatal cases karyotyped in this study. A few data sources related to Chinese population were also referenced, including the prevalence of DS in newborns from the “Report on birth defects in China, 2012” published by the Ministry of Health of China, the M/F ratio of DS identified from perinatal period by the National Center for Birth Defects in China [11], and the M/F ratio for all newborns in China estimated and published by the "Gender Statistics Highlights from 2012 World Development Report 2012" (available at: http://www-wds.worldbank.org/mwg-internal/de5fs23hu73ds/progress?id=uytrn6qWIQZwMXpjPYivvkoBeF5bJn9YxoppGjbyeMc,February 2012).

## Results

### Detection rates of Tri21

Karyotypes were successfully performed in 247,818 postnatal cases in our laboratory between January 1, 2011 and December 31, 2014, including 37,313 cases in 2011, 56,205 in 2012, 67,930 in 2013, and 86,370 in 2014 (S3 Table). In total, 7,133 cases with Tri21 were identified, representing a averaged detection rate of 2.88% (7,133/247,818) (Table 1, S3 Fig, and S3 Table). The detection rates for Tri21 for the years of 2011, 2012, 2013, and 2014 are 3.39%, 3.01%, 2.95%, and 2.52% respectively (S3 Table), showing significant decreases of the detection rates for Tri21 over the years except between 2012 and 2013 (S4 Table).

**Table 1:** Characterizations of Tri21.

Classifications and karyotypes	Numbers of Tri21 and percentages (%)				Gender	M/F Ratio	p-value
1. Standard Tri21 and non-contributory structural rearrangements	6,799 (95.32%)						
1a. Non-mosaic standard Tri21		6,671 (93.52%)				1.53	˂ 0.001
47,XX,+21				2,633 (36.91%)	F		
47,XY,+21				4,038 (56.61%)	M		
1b. Mosaic standard Tri21		96 (1.35%)				1.00	˃ 0.5
mos 47,XX,+21/46,XX				48 (0.67%)	F		
mos 47,XY,+21/46,XY				48 (0.67%)	M		
1c. Tri21 with non-contributory structural rearrangements		29 (0.41%)				1.46	˃ 0.1
ROBs			9 (0.13%)			2.00	˃ 0.1
46,XX,der(13;14)(q10;q10),+21				3 (0.04%)	F		
46,XY,der(13;14)(q10;q10),+21				6 (0.08%)	M		
Non-ROB structural rearrangements*			20 (0.28%)			1.50	˃ 0.1
				8 (0.11%)	F		
				12 (0.17%)	M		
2. Contributory structural rearrangements with Tri21	323 (4.53%)						
2a. Non-mosaic contributory ROBs with Tri21		316 (4.43%)					
der(l3;21)			22 (0.31%)			0.38	˂ 0.05
46,XX,der(13;21)(q10;q10),+21				16 (0.22%)	F		
46,XY,der(13;21)(q10;q10),+21				6 (0.08%)	M		
der(l4;21)			133 (1.87%)			1.11	˃ 0.1
46,XX,der(14;21)(q10;q10),+21				63 (0.88%)	F		
46,XY,der(14;21)(q10;q10),+21				69 (0.97%)	M		
46,XY,der(14;21)(q10;q10),t(6;12)(q21;q13),+21				1 (0.01%)	M		
der(l5;21)			6 (0.08%)			NT	NT
46,XX,der(15;21)(q10;q10),+21				2 (0.03%)	F		
46,XY,der(15;21)(q10;q10),+21				4 (0.06%)	M		
der(21;21)			148 (2.08%)			1.00	˃ 0.5
46,XX,der(21;21)(q10;q10)				74 (1.04%)	F		
46,XY,der(21;21)(q10;q10)				74 (1.04%)	M		
der(21;22)			7 (0.10%)			NT	NT
46,XX,der(21;22)(q10;q10),+21				2 (0.03%)	F		
46,XY,der(21;22)(q10;q10),+21				5 (0.07%)	M		
2b. Mosaic contributory ROBs with Tri21		7 (0.10%)				NT	NT
mos 45,XY,der(13;21)(q10;q10)/46,XY,der(21;21)(q10;q10)				1 (0.01%)	M		
mos 45,XY,der(14;21)(q10;q10)/46,XY,der(14;21)(q10;q10),+21				1 (0.01%)	M		
mos 45,XX,der(21;21)(q10;q10)/46,XX,der(21;21)(q10;q10)				1 (0.01%)	F		
mos 46,XX,der(21;21)(q10;q10)/46,XX				2 (0.028)	F		
mos 46,XY,der(21;21)(q10;q10)/46,XY				1 (0.01%)	M		
mos 45,XY,der(21;22)(q10;q10)/46,XY,der(21;22)(q10;q10),+21				1 (0.01%)	M		
2c. Non-ROB contributory structural rearrangements with Tri21		3 (0.04%)				NT	NT
47,XX,t(3;21)(q21;q22),+21				1 (0.01%)	F		
47,XX,t(9;21)(p11;p11),+21				1 (0.01%)	F		
47,XY,t(19;21)(p11;p11),+21				1 (0.01%)	M	2.67	˃ 0.1
3. Tri21 with additional aneuploidies	11 (0.15%)						
47,XY,der(21;21)(q10;q10),+mar				1 (0.01%)	M		
48,XX,+21,+mar				1 (0.01%)	F		
48,XXX,+21				1 (0.01%)	F		
48,XXY,+21				4 (0.06)	M		
48,XYY,+21				2 (0.03)	M		
mos 48,XX,+21,+mar/47,XX,+21				1 (0.01%)	F		
mos 48,XYY,+21/47,XY,+21				1 (0.01%)	M		
Total	7,133 (100%)					1.50	˂ 0.001
				2,858 (40.07)	F		
				4,275 (59.93)	M		

Notes: Tri21: trisomy 21; F: female; M: male; ROB: Robertsonian translocation, NT: not tested;

*The details are listed in S1 Table.

Group-1 comprises all standard Tri2 and their mosaics, and non-contributory Tri21 cases and their mosaics in which ROBs and other structural rearrangements are present, but not involved in the formation of Tri2 (Table 1). 6,799 Tri21 cases are classified in this group, representing 95.32% of the total Tri21 cases reported in this study, including 6,767 (93.52%) cases of non-mosaic standard Tri21, 96 (1.35%) cases of mosaic standard Tri21, 9 (0.13%) cases of non-contributory ROB Tri21 with a karyotype of [46,der(13;14),+21], and 23 (0.32%) cases of non-ROB non-contributory Tri21 with rare or private structural rearrangements (S1 Table).

Group-2 comprises all contributory Tri21 cases and their mosaics in which the formations of Tri21 are the consequence of ROBs or other structural rearrangements with the involvement of chromosome 21 (Table 1). In the current study, all 323 Tri21 cases classified in the group are contributory ROB Tri21 and their mosaics, representing 4.53% of the total Tri21 cases, including 148 (2.08%) cases with a karyotype of [46,der(21;21)], 133 (1.87%) cases of [46,der(14;21),+21], 22 (0.31%) cases of [46,der(13;21),+21], 7 (0.10%) cases of [46,der(21;22),+21], 6 (0.08%) cases of [46,der(15;21),+21], and 7 (0.10%) mosaic cases.

Group-3 comprises all Tri21 cases concurrent with additional aneuploidies and their corresponding mosaics (double aneuploidies). 11 (0.15%) Tri21 cases with non-ROB structural rearrangements were in this group, including 3 cases with an additional marker chromosome (two non-mosaic and one mosaic cases) and 8 cases with an additional sex chromosome (four cases of 48,XXY,+21, two 48,XYY,+21, one 48,XXX,+21, and one mosaic 48,XYY,+21/47,XY,+21) (S2 Table).

The 7,133 Tri21 cases were also stratified based on their ages and age groups (S4 and S5 Tables, S1 and S2 Figs). (1) 5,647 (79.17%) cases were newborns and infants, (2) 1,407 (19.73%) cases were children between the ages of 1 and 18 years old and only 21 cases in this group were between the ages of 14 and 18 years old, and (3) 79 (1.11%) cases were older than 18 years old. When non-mosaic Tri21 cases were compared with mosaic Tri21 in different age groups (S6 Table), non-mosaic Tri21 are more concentrated in newborns and infants (p ˂0.01) while mosaic Tri21 are more concentrated in children older than 1 year old and adults (p ˂0.01).

### M/F ratios in different types/subtypes of Tri21

The 247,818 postnatal cases karyotyped in this study were composed of 119,393 males, 128,405 females, and 20 cases with undetermined genders because these cases cannot be categorized as either male or female, such as 46,XX/45,XY, 45,X/46,X,+mar, etc. The overall M/F ratio is 0.93 (119,393/128,405) in this cohort of cases karyotyped, which is significantly lower than the M/F ratio of 1.20 for newborns in China ("Gender Statistics Highlights from 2012 World Development Report 2012") (P < 0.01), indicating that more female specimens have been referred to our laboratory for karyotype analysis.

The M/F ratio for the total 7,133 Tri21 cases is 1.50, significantly higher than the overall M/F ratio of 0.93, showing an excess of males with Tri21 in this cohort (p<0.001) (Table 1). The M/F ratios for most Tri21 subgroups with enough case numbers for statistical analysis were also compared with the overall M/F of 0.93 (Table 1). For the 6,671 cases with non-mosaic standard Tri21 (Group 1a), the M/F ratio is 1.53 (4,038 males/2,633 females), significantly higher than the overall M/F ratio of 0.93 (p<0.001). In contrast, the M/F ratio for the 96 cases with mosaic standard Tri21 (Group 1b) is 1.00, showing no statistical difference compared with the overall M/F ratio of 0.93 (P>0.5). Consistently, when compared with the non-mosaic standard Tri21, the mosaic standard Tri21 has a significantly lower proportion of males (P<0.05).

The M/F ratios for the 132 cases of [46,der(14;21),+21] and the 148 cases of [46,der(21;21),+21] are 1.11 and 1.00 respectively, showing no statistical differences with the overall M/F ratio of 0.93 (P˃0.1 and P˃0.5 respectively) (Group 2a). However, the M/F ratio of the 22 cases with [46,der(13;21),+21] is 0.375, showing an excess of females with Tri21 in this group (p<0.05).

Although a higher proportion of males were noticed in both Group-1c of Tri21 with non-contributory structural rearrangements and Group-3 of Tri21 with additional aneuploidies, there are no significant differences when their M/F ratios were compared with the overall M/F ratio of 0.93 separately due to their small sample sizes (p˃ 0.1). However, when the two subgroups were combined together, the M/F ratio (25 males and 14 females) is 1.79, significantly higher than the overall M/F ratio of 0.93 (p<0.05) (S2 Table). The justification for combining the two subgroups together is because that the concurrent existence of additional structural or numerical chromosomal abnormalities in the two subgroups is considered to be non-contributory to the formation of Tri21 [8,24].

The M/F ratios in different age groups of Tri21 were compared with the M/F ratio of 1.50 for the total 7,133 Tri21 cases instead of using the M/F ratio of 0.93 for the total cases karyotyped in this study (S4 and S5 Tables, S1 and S2 Figs). The M/F ratio of 1.51 in the age group of 0–1 year old and the M/F ratio of 1.53 in the age group of 1–18 years old are very similar to the M/F ratio of 1.50 for the total 7,133 cases with Tri21(p˃ 0.5). However, the M/F ratio in the age group older than 18 is 0.52 (27 males and 52 females) is significantly lower than the M/F ratio of 1.50, showing an excess of females with Tri21 in adults referred for karyotype analysis (p<0.001). When this group was further stratified, all 52 adult female Tri21 were younger than 29 years old and 14 of the 27 adult male Tri21 were older than 29 years old.

## Discussion

### Prenatal testing and elective termination of affected pregnancies might have contributed to the declines of positive Tri21 detection rates

The numbers of the karyotypes performed in our laboratory rose rapidly from 37,313 karyotypes successfully performed in 2011 to 86,370 in 2014 (S3 Table). However, the detection rates of Tri21 have declined significantly over the years except between the years of 2012 and 2013 (S3 and S4 Tables). We presume that the declines might reflect a possible impact of noninvasive prenatal testing (NIPT) applied rapidly in China and subsequent elective termination of affected pregnancies, although other factors, such as ascertainment bias over the years, cannot be ruled out. Based on surveillance data for DS from 1996 to 2011 carried out by the Chinese Birth Defects Monitoring Network, a recent study explored the impact of prenatal diagnosis and subsequent terminations on the birth prevalence of Down syndrome [11]. The data showed that the overall prenatal diagnosis of DS increased sharply from 12.98% in 2003 to 69.22% in 2011, and the termination rate increased from 17.65% to 94.47% and the high termination rate of affected pregnancies have led to 55% reduction in the overall DS perinatal prevalence in China. Increased termination rate of affected pregnancies were also observed in both Caucasian and other Asian populations where non-invasive prenatal screening and invasive prenatal diagnosis have been widely implemented in last twenty years [9,14,25,26]. Started from 2012, rapid expansion of NIPT by sequencing cell-free fetal DNA (cfDNA) in maternal plasma might also be a factor leading to the significant decline of the detection rate for Tri21 from 2.95% in 2013 to 2.52% in 2014 in this study (S3 and S4 Tables) [27,28]. This inference is supported by a recent report by Zhang et al who identified 1,107 possible T21 cases by sequencing cfDNA in maternal plasma for 146,958 pregnancies from January 1, 2012 to August 31, 2013, and the majority of these pregnancies ended due to either spontaneous or elective abortion [27]. We speculate that widespread applications of NIPT by sequencing cfDNA in maternal plasma combined with existing prenatal testing programs will further accelerate the decline of the postnatal detection rate for Tri21 in China.

### Cytogenetic Spectrum of Tri21

Although our findings were based on clinical cytogenetic diagnostics for a diversity of referrals, 95.32% of standard tri21, 4.53% of ROB Tri21, and less than 1% of other forms are very similar to the data in several large scale investigations about registered newborns with Tri21 in Caucasian populations and the comprehensively summarized data by Gardner et al in which 95% of DS are due to standard Tri21, 4% due to ROB Tri21, and 1% due to a number of rare forms of Tri21 [8,9,16,29]. These similarities strongly indicate that different racial/ethnic backgrounds might have played very limited influences to the genotypic variations of Tri21 if any of the influences might exist. We think those previously observed variations in different racial/ethnic backgrounds, such as, prevalence of Tri21 proportions of rare Tri21 karyotypes, M/F ratios, and clinical findings might have been influenced by differences from some non-biological factors such as policies, religions, cultures, social/economic levels, technique accessibilities, and data sources and qualities including sample sizes, ascertainment biases, analytical methods, etc. [1–17,20].

### There are more mosaic Tri21 in older children and adults

Mosaic Tri21is a condition in which an individual has two or more genetically distinct cell populations that originated from a single zygote and at least one of them contains Tri21 [30]. It is generally accepted that the zygotes in cases with mosaic Tri21 are initially trisomic for chromosome 21 and a second mitotic chromosomal segregation error gives rise to the euploid cell line. Mosaic Tri21/DS has been estimated to be approximately 1.3–5% of all Tri21/DS in western countries [18,30,31]. Of the 7,133 Tri21 identified in our study, 105 (1.47%) cases are mosaic Tri21. Comparing with the non-mosaic Tri21 group or total Tri21 cases in this cohort, there are significantly higher proportions of mosaic Tri21 in older children and adults (p˂0.001 and p˂0.01 respectively) (S6 Table). These results are consistent with previous observations that clinical features in individuals with mosaic Tri21 are generally milder and easily missed during perinatal period compared with those of children with non-mosaic DS [18,30,32]. For example, Bornstein et al. reported that fetuses with mosaic Tri21 showed a significantly lower frequency of ultrasound aberrations and screening test anomalies when compared to fetuses with non-mosaic Tri21 [33]. Based on a population study in Ireland, only 37.5% mosaic Tri21 cases were detected by clinical examination, compared to nearly 100% of the individuals with non-mosaic Tri21 [22]. We think the mosaic percentage of 1.47% in this study is a reasonable number to reflect the proportion of postnatal mosaic Tri21 in Chinese population for following reasons: (1) Based on the data from “Report on birth defects in China, 2012” that there are approximately 16 million newborns and the perinatal prevalence rate of DS is 1/580 in 2011 in China, we estimated that there are approximately 27,586 Tri2/DS newborns perinatally diagnosed each year in China. Thus, the 7,133 Tri21 cases (79.17% of them were newborns and infants) identified in our laboratory for a 4 year period from 2011–2014 is likely to represent a substantial proportion of the all Tri21 cases in China although our data may not be precisely applicable to the entire Chinese population. (2) Of the 7,133 Tri21 cases, 19.73% of them were children between the ages of 1 and 18 years old and 1.11% were older than 18 years old. The combined 20.84% in these two groups should have helped us to identify those mosaic Tri21 cases in which their clinical features are generally milder and easily missed [18,30,32]. (3) Based on the practice guidelines established by the American College of Medical Genetics released in 2010, clinical cytogenetic laboratories regularly analyze a minimum of 30 metaphases to rule out mosaic Tri21 if a patient is suspected to have mosaic Tri21 or one of the first 20 analyzed metaphases is Tri21 and analyzing 10 more metaphases are required. In our laboratory, a minimum of 60 metaphases for these scenarios was analyzed in order to enhance our ability to detect very low mosaicism of Tri21, such as, the case with mos 47,XY,+21 [2]/46,XY[58].

### Variable M/F ratios in different subtypes of Tri21

A higher proportion of males with non-mosaic Tri21 were consistently reported both prenatally and postnatally in all large scale studies for different racial/ethnic populations [8,11,17–19]. However, the genetic mechanisms leading to excessive males with non-mosaic Tri21 both prenatally and postnatally are unknown. Griffin et al. proposed that some of the excess of males among DS cases is attributable to a nondisjunctional mechanism in which the extra chromosome 21 preferentially segregates with the Y chromosome [34]. From studies based on newborn registries, the M/F ratios of Tri21 were 1.15 from the 6,424 livebirth with Tri21 from a pooled data of 10 sources [35], 1.20 from the New York State Chromosome Registry on over 10,000 cases of DS reported from 1977 to 1996 [17], 1.21 from the13,255 postnatal diagnoses of DS registered between January 1, 1989 and December 31, 2009 in England and Wales [8], and 1.30 from the 2,931 DS cases on the basis of the 1996 to 2011 surveillance data from Chinese Birth Defects Monitoring Network [11]. We speculate that these variations could be explained, at least partially, by background M/F ratio variations present in these populations. For example, the M/F ratio of 1.15 reported by Huether et al. was with a background ratio of 1.06 in births, the M/F ratio of 1.24 reported by Mutton et al. was with a background ratio of 1.05 in all births in the jurisdiction, and the M/F ratios of 1.30 reported by Deng et al. was with a background ratio of 1.20 in Chinese population based on the data from the "Gender Statistics Highlights from 2012 World Development Report 2012". In contrast to the overwhelming amounts of data based on newborn registries, information on the M/F ratios of Tri21 identified in clinical cytogenetics laboratories for diverse referral reasons are, however, still limited. The M/F ratio of 1.50 from our 7,133 Tri21 cases is significantly higher than these reported M/F ratios based on newborn registries cited above, including the M/F ratio of 1.30 for the perinatal Tri21 cases based on the surveillance data by the Chinese Birth Defects Monitoring Network (p<0.01) [11].

We noticed that Mandava et al. reported a M/F ratio of 1.84 based on the 1,572 DS cases identified in a clinical reference laboratory in India for diagnostic confirmation with chromosome analysis during 2000–2008 [19]. The reasons causing significant higher proportion of males with Tri21 identified in clinical reference laboratories are unknown. In our study, the M/F ratio of 1.51 in the group of newborns and infants and the ratio of 1.53 is in the age group of 1–18 years old, are not statistically different when compared with the M/F ratio of 1.50 for the total Tri21 cases in this study (S5 Table). Thus, a hypothesis that mosaic males with Tri21 are more likely to be positively selected over mosaic females with Tri21 for the relative likelihood of postnatal survival along with their increasing ages is less likely. However, it is interesting to notice there is an excessive proportion of adult females with Tri21 with a M/F ratio of 0.52 (p<0.001). More puzzled is that all 52 females in this group were younger than 29 years old, and 14 of the 29 males were older than 29 years old (S4 and S5 Tables). Considering the facts that the background M/F ratio of 1.20 in Chinese population and more severe gender inequality in rural areas of China, it might be a plausible explanation that females with Tri21 would have had more chances to get married and their subsequent reproduction difficulties in their early adulthood have led them to be referred for karyotype analysis.

In contrast to the significant higher proportion of males with Tri21, there are no sex bias in the 96 cases with mosaic standard Tri21 (M/F ratio of 1.00) or the 105 cases including all mosaic Tri21 (M/F ratio of 1.02). If the background M/F ratio of 1.20 in Chinese population is considered, the adjusted M/F ratio should be around 0.85, indicating that there are more female mosaic Tri21 cases in the general population. These results are very similar to the M/F ratio of 0.86 reported by hook et al., and the M/F ratio of 0.80 reported by Mutton et al. [8,17,24]. It is still a puzzle why mosaic Tri21 is excessive although some hypotheses were proposed [17]. Hulten et al. observed that females have a greater likelihood of being mosaics than males based on their findings that a low proportion of trisomy 21 cells was present in all eight fetal ovaries from normal women but not found in cells from fetal testes [36–38].

Our large data set in this study have enabled us to analyze the M/F ratio in several rare subgroups of Tri21. There are no significant sex bias in the subgroups of [46,der(14;21),+21] and [46,der(21;21),+21] whereas female Tri21 in the subgroup of [46,der(13;21),+21] is significantly higher with a M/F ratio of 0.38 (p<0.05). The reason causing the opposite M/F ratios in different subtypes of ROB Tri21 is unknown. Further studies to explore how variable genomic architectures present in these acrocentric chromosomes influence their nonrandom formation of ROBs and how these ROBs interact with other chromosomes by exerting their inter-chromosomal effects, especially chromosome Y, would provide some useful information to explain the M/F ratio differences [39,40]. The differences between the considerably high M/F ratio in non-mosaic Tri21 and the lower M/F ratios in all contributory ROB Tri21 subtypes should be the reflection of the heterogeneous underlying mechanisms leading to different Tri21 formations. Although in small sample sizes, the combined M/F ratio of 1.79 in non-contributory Tri21 groups including 9 cases of [46,der(13;14),+21] is similar to the 1.53 in non-mosaic Tri21, indicating that the Tri21 formation mechanisms between these two subgroups are similar but different from the contributory ROB Tri21. These observations also support the classification method for Tri21 karyotypes proposed by Mutton et al. based on presumed differences of the etiologies causing the formation of Tri21 [24]. The trend of an excess of Tri21+XXY compared with Tri21+XYY, or Tri21+XXX, was observed in this study, consistent with the previous reports by Morris et al [8]. Morris et al. also provided an explanation for this phenomena that about 50% of XXYs result from paternal nondisjunction of the sex bivalent and 50% from maternal nondisjunction, which contrasts with the XXX where almost all result from maternal nondisjunction and XYY which all result from paternal nondisjunction. Interestingly, the karyotype of [mos 48,XYY,+21/47,XY,+21] we identified is a novel finding and has not been reported earlier.

### Strengths and Weaknesses of the Study

Different from the several large scale studies on Caucasian populations based on the registered newborns, our study presented the largest collection of Tri21 in Chinese population based on extreme diversity of clinical findings and age differences. The different referral reasons and ages have enabled us to identify more Tri21/DS cases with atypical phenotypes, rare karyotypes, and low level of mosaicism. For example, the majority of the 23 non-contributory Tri21 cases caused by non-ROB structural abnormalities were not been reported previously (S1 Table). Although most of the results in our study agree with previous reports mainly based on Caucasian populations, some of our findings provide additional evidence to some controversial or unsolved questions in the previous reports as well as raise many new questions. For instance, why are there an unusually high proportion of males with non-mosaic Tri21 diagnosed in clinical setting than in newborns registries? Why are there a high proportion of females with mosaic Tri21? What are the underlying genetic mechanisms leading to opposite M/F ratios in some subtypes of ROB Tri21?

It is obvious that there are several weaknesses present in this study, such as (1) bias of ascertainments, (2) incomplete clinical information, especially for mosaic Tri21, which prevented us from carrying out genotype-phenotype correlation, and (3) some cases with structural abnormalities involving chromosome 21, such as dup(21) or add(21), were excluded from this study due to their uncertain connections with the clinical findings in these cases.

In conclusion, our large collection of Tri21 cases allowed us to determine the occurrence frequency of the different karyotypes of Down syndrome in China. A diversity of clinical findings as well as wide ranges of ages in these Tri21 cases enabled us to established several hypotheses. A long list of rare or private karyotypes identified in this study expanded the genotype spectrum in individuals with Tri21.

## Supporting Information

S1 Fig Male/female ratios in different age groups. (TIF)

S2 Fig Cases distribution in different age groups. (TIFF)

S3 Fig Proportions of different types of Tri21. (TIF)

S1 Table Tri21 with non-contributory structural rearrangements excluding ROBs. (DOCX)

S2 Table Tri21 with non-contributory chromosomal abnormalities. (DOCX)

S3 Table Postnatal karyotype analyses performed over the years in this study. (DOCX)

S4 Table Comparison of the detection rates of Tri21 year by year. (DOCX)

S5 Table Comparison of M/F ratios in different age groups. (DOCX)

S6 Table Mosaic and non-mosaic Tri21 among different age groups. (DOCX)
