# Supplementary figures and images for: Postnatal Identification of Trisomy 21: An Overview of 7,133 Postnatal Trisomy 21 Cases Identified in a Diagnostic Reference Laboratory in China
Source: PLoS One. 2015 Jul 15;10(7):e0133151. doi: 10.1371/journal.pone.0133151 (PMC4503670; doi:10.1371/journal.pone.0133151)

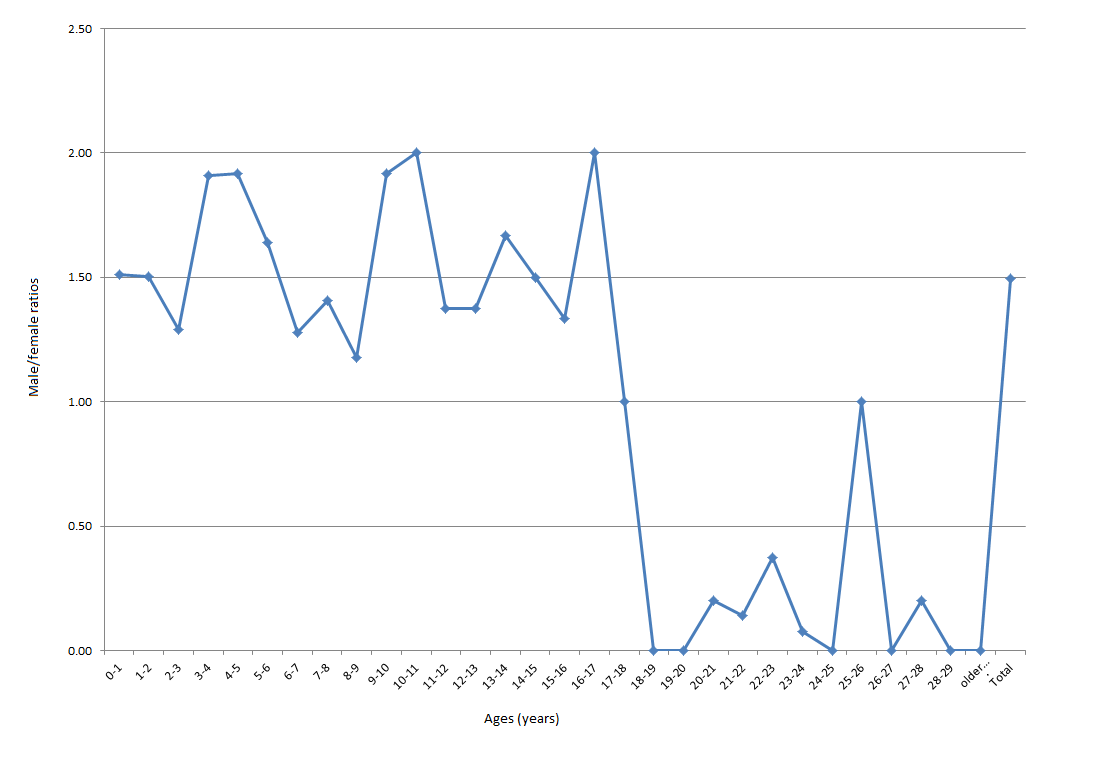

Supplement: S1 Fig — (TIF) [file pone.0133151.s001.tif]

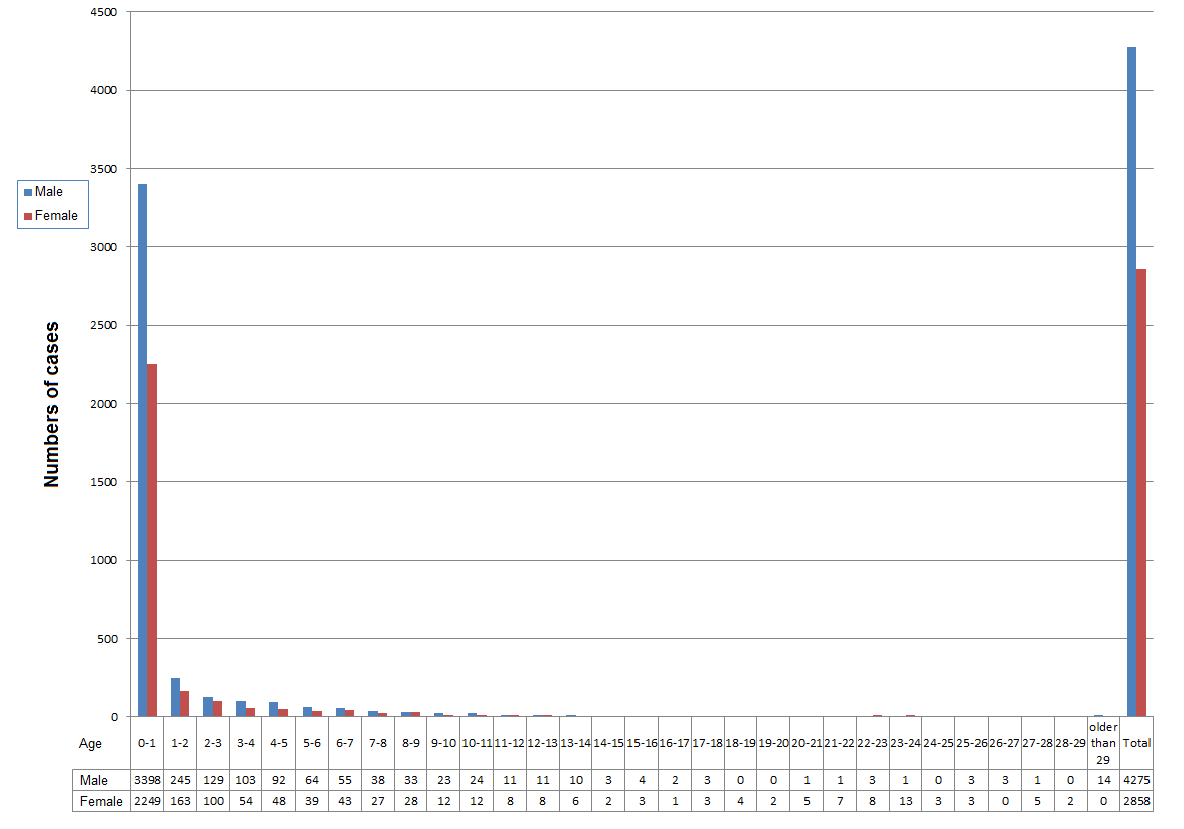

Supplement: S2 Fig — (TIFF) [file pone.0133151.s002.TIFF]

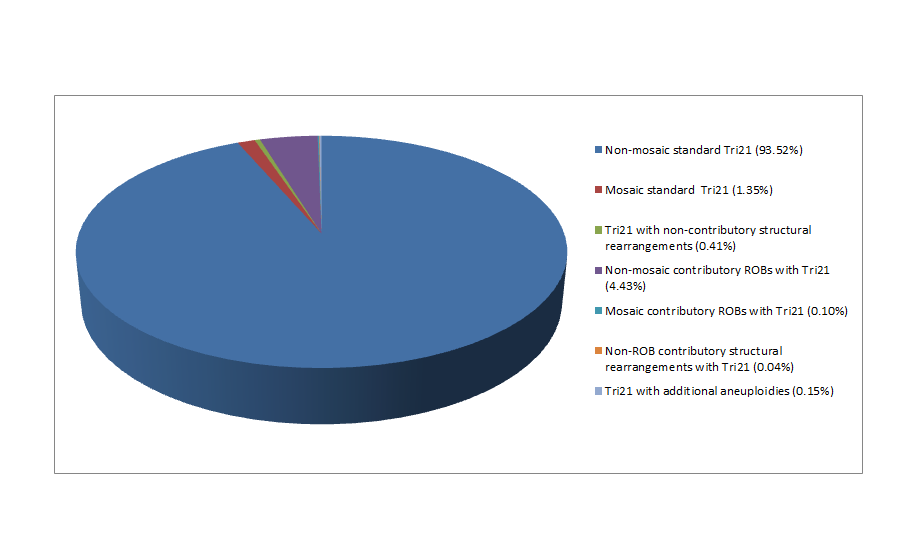

Supplement: S3 Fig — (TIF) [file pone.0133151.s003.tif]
