# Supplementary material for: Postnatal Identification of Trisomy 21: An Overview of 7,133 Postnatal Trisomy 21 Cases Identified in a Diagnostic Reference Laboratory in China
Source: PLoS One. 2015 Jul 15;10(7):e0133151. doi: 10.1371/journal.pone.0133151 (PMC4503670; doi:10.1371/journal.pone.0133151)
Supplement: S1 Table — (DOCX) [file pone.0133151.s004.docx]

| **S1 Table. Tri21 with non-contributory structural rearrangements excluding ROBs** | | |
| --- | --- | --- |
| **Karyotypes** | **Numbers** | **Gender** |
| **47,XY,add(5)(p15.1),+21** | **1** | **M** |
| **47,X,i(X)(q10),+21** | **1** | **F** |
| **47,XY,ins(10;?)(q11.1;?),+21** | **1** | **M** |
| **47,XY,inv(2)(p11.2q13),+21** | **1** | **M** |
| **47,XY,inv(8)(p11.2q21.2),+21** | **1** | **M** |
| **47,XX,inv(11)(p11q25),+21** | **1** | **F** |
| **47,XX,t(1;11)(p31;q23),+21** | **1** | **F** |
| **47,XY,t(2;4)(q21;p14),+21** | **1** | **M** |
| **47,XX,t(2;4)(q33;q25),+21** | **1** | **F** |
| **47,XY,t(2;5)(p13;q11.2),+21** | **1** | **M** |
| **47,XX,t(2;7)(q13;q22),+21** | **1** | **F** |
| **47,XX,t(2;9)(p21;p24),+21** | **1** | **F** |
| **47,XX,t(2;19)(p13;q13.1),+21** | **1** | **F** |
| **47,XY,t(4;5)(q31;q34),+21** | **1** | **M** |
| **47,XY,t(4;15)(q31.3;q26.1),+21** | **1** | **M** |
| **47,XY,t(5;11)(q13;q13),+21** | **1** | **M** |
| **47,XX,t(6;11)(q27;q21),+21** | **1** | **F** |
| **47,XY,t(7;16)(p13;p13.1),+21** | **1** | **M** |
| **47,XY,t(11;13)(p15;q22),+21** | **1** | **M** |
| **47,XY,t(11;22)(q23;q11.2),+21** | **1** | **M** |
