# Supplementary material for: Postnatal Identification of Trisomy 21: An Overview of 7,133 Postnatal Trisomy 21 Cases Identified in a Diagnostic Reference Laboratory in China
Source: PLoS One. 2015 Jul 15;10(7):e0133151. doi: 10.1371/journal.pone.0133151 (PMC4503670; doi:10.1371/journal.pone.0133151)
Supplement: S2 Table — (DOCX) [file pone.0133151.s005.docx]

| **S2 Table. Tri21 with non-contributory chromosomal abnormalities** | | |  |  |  |
| --- | --- | --- | --- | --- | --- |
| **Karyotype** | **Number** | | **Gender** | **M/F Ratio** | **p-value** |
| **1c. ROBs** | **9** |  |  | **2.00** | **˃ 0.1** |
| **46,XX,der(13;14)(q10;q10),+21** |  | **3** | **F** |  |  |
| **46,XY,der(13;14)(q10;q10),+21** |  | **6** | **M** |  |  |
| **1c. Non-ROB structural rearrangements*** | **20** |  |  | **1.50** | **˃ 0.1** |
|  |  | **8** | **F** |  |  |
|  |  | **12** | **M** |  |  |
| **3. Aneuploidies** | **10** |  |  | **2.33** | **˃ 0.1** |
| **48,XX,+21,+mar** |  | **1** | **F** |  |  |
| **48,XXX,+21** |  | **1** | **F** |  |  |
| **48,XXY,+21** |  | **4** | **M** |  |  |
| **48,XYY,+21** |  | **2** | **M** |  |  |
| **mos 48,XX,+21,+mar/47,XX,+21** |  | **1** | **F** |  |  |
| **mos 48,XYY,+21/47,XY,+21** |  | **1** | **M** |  |  |
| **Total** | **39** |  |  | **1.79** | **˂ 0.05** |
|  |  | **14** | **F** |  |  |
|  |  | **25** | **M** |  |  |
| **Note: * The details are listed in S1 Table.** |  |  |  |  |  |
