# Supplementary material for: Postnatal Identification of Trisomy 21: An Overview of 7,133 Postnatal Trisomy 21 Cases Identified in a Diagnostic Reference Laboratory in China
Source: PLoS One. 2015 Jul 15;10(7):e0133151. doi: 10.1371/journal.pone.0133151 (PMC4503670; doi:10.1371/journal.pone.0133151)
Supplement: S3 Table — (DOCX) [file pone.0133151.s006.docx]

| **S3 Table. Postnatal karyotype analyses performed over the years in this study.** | | | | | | |
| --- | --- | --- | --- | --- | --- | --- |
| **Years** | **Total casess** |  | **Gender** | **Male/Female Ratio** | **Tri21*** | **Detection rate of Tri21 (%)** |
| **2011** | **37,313** |  |  | **0.94** | **1,265** | **3.39** |
|  |  | **19,257** | **Female** |  |  |  |
|  |  | **18,048** | **Male** |  |  |  |
|  |  | **8** | **ND**** |  |  |  |
| **2012** | **56,205** |  |  | **0.91** | **1,689** | **3.01** |
|  |  | **29,387** | **Female** |  |  |  |
|  |  | **26,812** | **Male** |  |  |  |
|  |  | **6** | **ND**** |  |  |  |
| **2013** | **67,930** |  |  | **0.94** | **2,005** | **2.95** |
|  |  | **35,002** | **Female** |  |  |  |
|  |  | **32,927** | **Male** |  |  |  |
|  |  | **1** | **ND**** |  |  |  |
| **2014** | **86,370** |  |  | **0.93** | **2,174** | **2.52** |
|  |  | **44,759** | **Female** |  |  |  |
|  |  | **41,606** | **Male** |  |  |  |
|  |  | **5** | **ND**** |  |  |  |
| **Total** | **247,818** |  |  | **0.93** | **7,133** | **2.88** |
|  |  | **128,405** | **Female** |  |  |  |
|  |  | **119,393** | **Male** |  |  |  |
|  |  | **20** | **ND**** |  |  |  |
| **Notes: *Tri21: trisomy 21; **ND: not be able to be determined genotypically and/or phenotypically.** | | | | | | |
