# Supplementary material for: Postnatal Identification of Trisomy 21: An Overview of 7,133 Postnatal Trisomy 21 Cases Identified in a Diagnostic Reference Laboratory in China
Source: PLoS One. 2015 Jul 15;10(7):e0133151. doi: 10.1371/journal.pone.0133151 (PMC4503670; doi:10.1371/journal.pone.0133151)
Supplement: S4 Table — (DOCX) [file pone.0133151.s007.docx]

| **S4 Table. Comparison of the detection rates of Tri21 year by year.** | | | |
| --- | --- | --- | --- |
| **Years** | **Chi square** | **p value** |  |
| **2011/2012** | **10.2** | **˂0.01** |  |
| **2011/2013** | **14.45** | **˂0.001** |  |
| **2011/2014** | **69.28** | **˂0.001** |  |
| **2012/2013** | **0.28** | **˃0.5** |  |
| **2012/2014** | **29.11** | **˂0.001** |  |
| **2013/2014** | **25.79** | **˂0.001** |  |
