# Supplementary material for: Postnatal Identification of Trisomy 21: An Overview of 7,133 Postnatal Trisomy 21 Cases Identified in a Diagnostic Reference Laboratory in China
Source: PLoS One. 2015 Jul 15;10(7):e0133151. doi: 10.1371/journal.pone.0133151 (PMC4503670; doi:10.1371/journal.pone.0133151)
Supplement: S5 Table — (DOCX) [file pone.0133151.s008.docx]

| **S5 Table. Comparison of M/F ratios in different age groups** | | | | | |  |  |  |
| --- | --- | --- | --- | --- | --- | --- | --- | --- |
| **Age group** | | **Number (%)** | | | **Gender** | **M/F ratio** | | **p value*** |
| **0-1** |  | **5647 (79.17%)** |  |  |  | **1.51** |  | **˃ 0.5** |
|  |  |  |  | **2,249** | **F** |  |  |  |
|  |  |  |  | **3,398** | **M** |  |  |  |
| **1-18** |  | **1407 (19.73%)** |  |  |  | **1.53** |  | **˃ 0.5** |
|  |  |  |  | **557** | **F** |  |  |  |
|  |  |  |  | **850** | **M** |  |  |  |
| **˃ 18** |  | **79 (1.11%)** |  |  |  | **0.52** |  | **˂ 0.001** |
|  | **˃ 18-29** |  | **48** |  |  |  | **0.14** | **˂ 0.001** |
|  |  |  |  | **52** | **F** |  |  |  |
|  |  |  |  | **13** | **M** |  |  |  |
|  | **˃ 29** |  | **14** |  |  |  | **NA** |  |
|  |  |  |  | **0** | **F** |  |  |  |
|  |  |  |  | **14** | **M** |  |  |  |
| **Total** |  | **7133 (100%)** |  |  |  | **1.50** |  |  |
|  |  |  |  | **2,858** | **F** |  |  |  |
|  |  |  |  | **4,275** | **M** |  |  |  |
| **Notes: * compared with the M/F ratio of 1.50 for the total Tri21 cases in this study.** | | | | | | | | |
