# Supplementary material for: Postnatal Identification of Trisomy 21: An Overview of 7,133 Postnatal Trisomy 21 Cases Identified in a Diagnostic Reference Laboratory in China
Source: PLoS One. 2015 Jul 15;10(7):e0133151. doi: 10.1371/journal.pone.0133151 (PMC4503670; doi:10.1371/journal.pone.0133151)
Supplement: S6 Table — (DOCX) [file pone.0133151.s009.docx]

| **S6 Table. Mosaic and non-mosaic Tri21 among different age groups.** | | | | | | |  |  |
| --- | --- | --- | --- | --- | --- | --- | --- | --- |
| **Age (year)** | **Mosaic Tri21** | | **Non-mosaic Tri21** | | **Total Tri21** | | **P Value** | **Group comparison** |
|  | **Number** | **Percentage** | **Number** | **Percentage** | **Number** | **Percentage** |  |  |
| **0-1** | **69** | **65.71** | **5,578** | **79.37** | **5,647** | **79.17** | **˂0.01** | **between 0-1 and 1-18** |
| **˃1-18** | **30** | **28.57** | **1,377** | **19.59** | **1,407** | **19.73** | **˂0.001** | **between 0-1 and ˃ 18** |
| **˃ 18** | **6** | **5.71** | **73** | **1.04** | **79** | **1.11** | **˂0.01** | **between 1-18 and ˃ 18** |
| **Total** | **105** | **100** | **7,028** | **100** | **7,133** | **100** |  |  |
